# Detroit Academy of Medicine

**Published:** 1880-08

**Authors:** 


### DETROIT ACADEMY OF MEDICINE.

---

Dr. Bradley reported a case of melanotic sarcoma of right eye. Operation.

Dr. Noyes—It is some time ago, but still I remember the case distinctiy, when I was called in consultation. We both considered the tumor malignant, and it was on that account the eye was removed. The success in these cases is much more sure if extirpation is made before the tumor has broken through the tunics of the eye.

Dr. Connor—I remember seeing a case in the Charity Hospital while I was a student, and I afterwards made a post-mortem examination of the case. The patient's body was studded over with any number of melanotic tumors.

The disease began in the right eye with great pain, but the eye was never removed, and after a while tumors began to develop themselves over the body, all of them showing the characteristic color. The patient lived a short time and died.

Dr. Carsten—I have here a very peculiar tumor taken from a girl fifteen years of age. She was well developed and apparently in good health. The growth occupied a portion of the mons veneris and hung down in front of the vagina, and looked like a penis. Some of the people who saw it thought the girl was a hermaphrodite. The tumor resembled, to a certain extent, elephantiasis, which is common to these parts. I removed it readily with a knife and brought the edges of the skin together, and left no scar. The tumor is, as you see, fibro-cystic in character, and under the microscope is seen to contain large quantities of epithelial elements. It is of interest, owing to its peculiar shape and the position it occupied.

Dr. Connor exhibited to the Society Risley's optometer, showing the facility with which it enabled one to measure and record the field of vision of either eye, to detect and accurately measure any degree of astigmatism, of hypermetropia, of myopia, or of presbyopia. While other means enabled the oculist to reach the same results, this apparatus certainly enabled him to make more accurate observations with a saving of time and an increase of comfort to both his patient and himself. Beyond question it is the most perfect apparatus of its kind now before the profession.

Dr. Noyes—The great value of the intrument is seen at a glance. It records exactly the field of vision, the amount of defect in the eyes and many other points that are essential to a correct examination. The instrument is not new, but has been so expensive that it did not come into general use. I am glad that Dr. Connor has procured one, and I am sure he will find it a great help in diagnosis. I think that he will have no reason to regret having invested in so valuable and expensive an instrument.

Dr. Noyes—I will report a case of a little child six years old, who received a stab in the right eye with a large knife in the hands of a playmate. The cut was fully a quarter of an inch long in the sclera, and involving the ciliary region of the eye. A week after the accident the little patient was brought to me. No bandage having been applied by the physician who had had the case in charge, the wound was found to be gaping open with a portion of the iris and vitreous protruding through it. I removed the piece of iris which was protruding from the wound and applied a compress bandage; that was about all I did. I had the child under observation only a week, as the parents were not able to stay longer in the city. The inflammation subsided to a great extent, and no symptoms of irritation have appeared in the fellow eye. I need hardly say, that all vision was lost in the injured eye. I gave the mother directions to continue the bandage, and that if any weakness or tenderness appeared in the well eye to return immediately to me, as it might become necessary in that case to enucleate the injured eye. I have not heard from them since, and I am to suppose that no trouble has arisen For cosmetic reasons, and for the support which the eye gives the lids, it is always best to retain the injured organ in such a case if it is possible to do so without incurring too great a risk. It need scarcely be said here that in such an accident as this the first thing, almost, a physician should do would be to close the lids and apply a compress bandage.

Dr. Carrier—Does the eye retain its shape and position in those cases where the optic nerve has been divided?

Dr. Noyes—Yes; the eye retains nearly its shape and size, and is quite successful in preventing the sympathetic difficulties for which eyes have hitherto been removed. The operation is becoming quite popular

Dr. Bradley—Would not the operation be more apt to be followed by suppuration and inflammation of the cellular tissues posterior to the eye? Would not the hemorrhage be great and hard to suppress in some cases?

Dr. Noyes—Thus far the operation has been quite successful. Of course there are cases where suppuration and hemorrhage might occur, which would make the case difficult to handle.

Dr. McGraw—I have a class of students at St. Mary's Hospital, in the wards. Among other things I have them each get a thermometer and take the temperature of the surgical patients. After examining several patients it was found that there was a difference of several degrees in the thermometers used, some registering more and some less. We found that they all registered correctly the normal temperature, but when using them on the same patient at the same time, in the same position, it was found that there was a disparity of several degrees, no two registering alike. If this is the case with all thermometers used by medical men throughout the country, I can hardly see how we can place any reliance on the statements we see in medical journals where reports of cases of high temperatures are given. We often see reports of cases where the temperature ranges as high as 106° or 107° F. that recover under treatment. Probably these physicians were using high temperature thermometers, while other physicians lose patients with comparatively low degrees of fever. These differenoes are, no doubt, due to incorrect registration of the thermometers used. It is something that ought to be looked into.

